# Localised learning: mobilising belonging among mature-aged students in low socio-economic status regional and remote areas

**DOI:** 10.1007/s10734-022-00877-x

**Published:** 2022-05-31

**Authors:** Nicole Crawford, Lara McKenzie

**Affiliations:** 1 National Centre for Student Equity in Higher Education (NCSEHE), Curtin University, Perth, Australia; 2 Anthropology and Sociology, The University of Western Australia, Perth, Australia

**Keywords:** Higher education, Mature-aged students, Regional and remote, Low socio-economic status (SES), Belonging, Learning support

## Abstract

The higher education participation and success rates of students in low socio-economic status (SES), regional, rural, remote, and isolated areas – who often attend university later in life – is a persistent concern in Australia and beyond. This article focuses on mature-aged students in low SES, regional and remote areas in Tasmania, Australia, proposing that universities harness local belonging when providing learning opportunities. It draws on a thematic analysis of 19 semi-structured interviews with current and prospective university students, and community stakeholders. The study identifies time and place-based barriers to studying on campus: students’ commitments outside of university; and geographical, cultural, and financial challenges. However, existing local infrastructure, such as libraries, create opportunities for face-to-face interactions and learning support for students who study online in their regional or remote communities, provided by staff and local volunteers. These barriers and solutions are discussed using the concept of ‘belonging’, framed spatially and culturally. Current literature on regional and remote higher education students tends to emphasise ‘not belonging’ *in relation to* distant urban or metropolitan spaces. We argue that ‘belonging’ can be fostered in local spaces with local people. Utilising ‘untapped’ local learning support and existing physical spaces mitigates geographical, cultural, and financial challenges, and provides academic and emotional support. We propose a coordinated network of physical study places and local people, including: regional ‘satellite’ campuses; regional study hubs; local public libraries; and schools, where online students can be supported, connected, and engaged in their studies whilst located in regional and remote communities.

## Introduction

The difficulties of engaging in education in regional, rural, remote, and isolated areas have been well documented in Australia and internationally (Crawford, 2021; Currie et al., 2007; Halsey, 2018; Johns et al., 2016; Kenyon, 2011; Power et al., 2019; Regional Education Expert Advisory Group, 2019). Australia, with its low population density across large areas of the country, provides a notable case of the impact of ‘geographic dispersion’ (Bradley et al., 2008, p. 111). While most of the country’s 42 universities are located in major cities, ten have their main campus in regional cities. Furthermore, a number of smaller ‘satellite’ campuses and study hubs are located in regional areas. Yet prospective and current university students still face unique challenges when separated from ‘main’ campuses by large distances or financial, personal, and geographical barriers. This is especially the case for mature-aged and low socio-economic status (SES) students. In Australia, higher proportions of mature-aged undergraduate university students are in, or from, regional and remote, and low SES areas, and study online and part-time, compared to students in major cities (Crawford, 2021, pp. 26–29).

This article focuses on the needs of mature-aged students located in low SES, regional and remote areas in Tasmania, Australia. In Australia, people living in low SES, regional and remote areas are less likely to have post-secondary school qualifications compared to their counterparts in major cities (Australian Bureau of Statistics, 2020a, b). They are also more likely be studying as mature-aged students (Crawford, 2021, pp. 26–27). Tasmania, the smallest state in Australia, and an island, has one university, with several campuses located in the capital city, Hobart, and smaller campuses in regional centres. It also has one of the lowest levels of educational attainment in Australia (Australian Bureau of Statistics, 2020a). In 2020, Tasmania was lowest of the Australian states and territories on the Australian Digital Inclusion Index (ADII), which measures digital ability, access to, and affordability of the internet (Thomas et al., 2020, p. 41). Tasmania is, thus, a useful site for considering the intersecting and compounding challenges of regional/remote geographical locations, low SES, and studying as a mature-aged student with parenting, work and community commitments.

We explore students’ challenges through semi-structured interviews with community stakeholders, and current and prospective mature-aged students. Our focus is on time and place-based barriers to studying on major campuses, including non-university commitments and geographical, cultural, and financial challenges. This includes students’ sense of distance to/from and ‘not belonging’ on campus. Yet solely emphasising feelings of exclusion, isolation, and distance, as has been common in previous approaches (Jury et al., 2017; Regional Education Expert Advisory Group, 2019), can unfortunately render these regional and remote students problematic, rather than recognising their strengths. Through the lenses of ‘belonging’ and ‘distance’, and adopting a ‘rural standpoint’ (Roberts, 2014), we propose a series of engagement and support strategies that utilise local people and infrastructure to mitigate the challenges of studying in regional and remote areas. Our findings prompt us to re-consider ‘distance’ as measuring distance to/from urban areas. Instead of focusing solely on students’ experiences of not having access to or belonging on urban and major regional campuses, we highlight the uncharted potential of local spaces and belongings.

## Background

### Defining the terms

There is no standard definition of mature-aged students in higher education in Australia. For the purposes of this study, a mature-aged student is defined as being twenty-one years of age or older at commencement of their undergraduate course. Socio-economic status is defined using the Australian Bureau of Statistics (2018a) Socio-Economic Indexes for Areas (SEIFA) Index of Education and Occupation (IEO) scores, with areas scoring from one to twenty-five per cent being considered low SES.^1^ Different terms are used in the literature to define and classify students who come from or reside in regional, rural, remote, or isolated areas in Australia. In this article, the term ‘regional and remote’ is used to encompass regional, rural, remote, and isolated. It is defined according to the Australian Statistical Geography Standard (ASGS): Remoteness Structure (Australian Bureau of Statistics, 2018c), which divides Australia into five categories: major city (RA1); inner regional (RA2); outer regional (RA3); remote (RA4); and very remote (RA5). Thus, the term ‘regional and remote’ includes inner regional, outer regional, remote, and very remote.^2^ All of Tasmania is categorised as regional or remote, and many areas of the state are low SES (Australian Bureau of Statistics, 2018a, c).

### The equity context

In Australia, universities and governments have implemented strategies and targets to widen participation in higher education, particularly among students from six designated equity groups.^3^ In recent years, there has been growth in the participation rates of domestic undergraduate students from some of these equity groups: for example, students from low SES areas and Aboriginal and Torres Strait Islander students (Koshy, 2020). However, there have been lower rates of enrolment for regional and remote students (Koshy, 2020; Pollard, 2018), and these students’ completion rates are lower than their metropolitan counterparts (Pollard, 2018), indicating an urgent need to consider their educational experiences (Halsey, 2018; Regional Education Expert Advisory Group, 2019). Moreover, studies show that equity-group categories tend to overlap, reflecting multiple and compounding challenges for many students (Cassells et al., 2017; Willems, 2010). Further characteristics of regional and remote, mature-aged students include having substantial work and parenting commitments, being first in their family to study at university, combined with studying part-time and online (Crawford, 2021; Crawford & Emery, 2021).

Mature-aged students have been less of a focus than their school-leaver counterparts in national reviews of regional and remote education (Halsey, 2018; Regional Education Expert Advisory Group, 2019); they are, nevertheless, an important cohort, making up large numbers of university enrolments.^4^ They often want or need to remain in their communities while studying (Johns et al., 2016). They tend not to relocate for further studies; rather, they study from the regional or remote areas where they live, work, and parent (Crawford, 2021). Despite their other responsibilities and barriers in adjusting to university, once enrolled mature-aged students tend to do as well as, if not better than, their younger counterparts (Craft, 2019; Tones et al., 2009).

To increase access and participation of students in equity groups, most Australian universities conduct outreach activities. These efforts typically focus on primary and secondary school students, rather than mature-aged students. They also often ignore the ways that the country’s higher education system is built with young, high SES, urban, on-campus students’ comfort and belonging in mind, and presume that these students are the norm (Crawford, 2021). Regional and remote, low-SES, mature-aged students tend to be treated as a problem or challenge, whose lower participation rates need to be ‘fixed’ by allowing them to attend and integrate more effectively into (largely metropolitan) university campuses. However, the development of less centralised university campuses and services can and has begun to challenge this view.

### Regional and remote study facilities

While all students are eligible to access the centralised support provided by their universities, regional and remote students cannot necessarily do so easily if support is provided at the ‘main’ city campus. Universities headquartered in regional areas, as well as universities with regional ‘satellite’ campuses, tend to be better equipped to provide nearby regional students with face-to-face support and library services (Craft, 2019; Rossi & Goglio, 2020). Where they exist, these satellite campuses explicitly aim to promote local accessibility in regional and outer urban areas, and often seek to enrol low SES students (Craft, 2019, p. 1374).

Yet, to be truly effective, higher education scholars and practitioners have argued that providers also need to support, encourage, and build ‘partnerships with local communities, providers in other sectors of education, [and] businesses and industry’ (Bradley et al., 2008, p. 111). In the UK context, Elliott (2018, 2019) describes a ‘partnership’ approach between a university and a not-for-profit private provider – implemented for mature-aged female students in coastal, rural, and isolated communities of south-west England – that enables students to learn locally while they work and parent, and does not require them to relocate for their studies. Cross-institutional collaborations and partnerships can also involve the use of shared facilities and resources. Local libraries, schools, and regional study hubs have the potential to provide study spaces and student support outside of the institution in which students are enrolled. For instance, public libraries provide non-intimidating spaces where university students, studying online in regional and remote areas, can study and access the internet and computers, as well as receive support from librarians (Behr & LaDell-Thomas, 2014; Howlett et al., 2017; Power et al., 2019).

In the Australian context, the ‘Open Universities Australia (OUA) Connect Library Program’ was one initiative that linked online university students with local libraries (Stone, 2013), and the collaboration between the University Preparation Program^5^ at the University of Tasmania (UTas) and Libraries Tasmania was another (Jarvis et al., 2013). However, with changes in personnel, both initiatives faded. Local schools have likewise been proposed as ‘community hubs’ for education (Gore et al., 2019, p. 66; Pollard 2018, p. 38). In recent years, the Australian Government Department of Education, Skills and Employment (2020b) has committed to funding regional study hubs around Australia, referred to as the ‘Regional University Centres’ program. Designed for students studying online and living in regional and remote areas, these study hubs are physical spaces with reliable internet and computers, and academic and pastoral support. Current initiatives for regional and remote students vary hugely across space and over time. While many schemes have focused on providing one-on-one, practical, or social support to students, they have done so with varying degrees of funding and many interventions have been short-term.

### Distance and ‘not belonging’

While researchers have explored the experiences of regional and remote university students, less attention has been paid to the impact of geographical distance, transport, and travel on these students (Currie et al., 2007; Kenyon, 2011). Issues of geography and finance were highlighted by a recent survey of 18,584 Australian university students (Universities Australia, 2018, p. 64), which found that 62.7 per cent of low SES domestic undergraduates and 64.2 per cent of regional domestic undergraduates often worried about their finances. Less than half of both of these groups reported having ‘no problem with the cost of travel in attending classes’, indicating that the majority have difficulty paying for travel.

Some higher education research shows how exclusion and distance overlap. Kenyon (2011, p. 764) explores transport and ‘social exclusion’ in UK higher education, finding that transportation difficulties, costs, and distance are major access barriers for students. She highlights the problems of transport for people without cars, with young children, and those working or travelling outside office hours. Spatial barriers encompass both ‘the physical proximity of activities and environmental features, which influence the accessibility of non-motorised and motorised mobility’ (Kenyon, 2011, p. 765). Kenyon (2011) also suggests that low SES students, and students who do not live close to campus, regularly miss out on learning, career, and social opportunities, and have concerns about the safety, reliability, and affordability of travel to and from campus. These experiences mean that universities are seen as places that are difficult to get to (Gore et al., 2019; Webb et al., 2015).

Jury et al. (2017, p. 26) report that low SES students often feel they ‘do not belong’ in the unfamiliar spaces of university campuses. They outline what they call students’ ‘psychological barriers’, resulting from their cultural distance from universities. They describe these barriers as negatively impacting students’ emotional experiences of distress or wellbeing, their identities, senses of belonging, self-perceptions, and motivations (p. 25). Other researchers warn against focusing solely on people’s ‘individual sense[s] of belonging’ (Lähdesmäki et al., 2016, p. 241), and ignoring the contexts in which they are embedded (Antonsich, 2010, p. 653). It is argued that any analysis of belonging must examine the physical and the social, as well as the personal (May, 2011). In keeping with this approach, we analyse belonging in relation to distance and local spaces.

### Conceptualising ‘belonging’ in higher education research

Our use of ‘belonging’ is informed by a critical understanding of urban and regional/remote separation or ‘distance’. Here, Roberts’ (2014) ‘rural standpoint’ epistemology is useful. He argues that much research purporting to be centred on the rural ‘tacitly assume[s] a metropolitan norm, where the point of difference is the context of rural’ (Roberts, 2014, p. 135). In the case of regional and remote students, for instance, ‘distance’ is invoked in terms of ‘distance from’ major (usually urban) campuses. The same goes for scholars’ focus on regional and remote students’ ‘belonging’ – and, indeed, the very terminology of ‘regional’ and ‘remote’ – which regularly emphasises non-belonging on major campuses (Jury et al., 2017; Regional Education Expert Advisory Group, 2019), rather than local iterations of belonging. We show that by looking beyond ideas of regional and remote separation and distance, and recentring these communities and the people that ‘belong’ to them, new possibilities for locally embedded learning become evident.

Here, a deeper look at the concept of belonging is required. May (2011, p. 368) describes belonging as ‘a sense of ease with oneself and one’s surroundings’, established through relations and interactions with other people. Massey (2011) takes a different approach, and asks to whom places belong. Nonetheless, belonging tends to be attached to people and power as well as place (Yuval-Davis, 2006). It is regularly described in spatial terms, referencing places, their boundaries (Lähdesmäki et al., 2016), and the emotional connections they inspire (May, 2011). Yet belonging is not fixed or static. It can shift over time and across spaces, and it is not simply located in one place (Gravett & Ajjawi, 2021). Moreover, feelings and realities of *not* belonging can emerge and become firmly attached to particular places: far-off metropolitan universities, for instance.

Indeed, as May (2011, p. 369) notes, ‘not everyone is allowed to belong’, and belonging is not only about individual people’s feelings. As alluded to above, it is common for those using the concept to focus on experiences and feelings of *not* belonging, particularly among ‘ethnic, racial, or national minorities and/or otherwise marginalized groups’ (Antonsich, 2010; Lähdesmäki et al., 2016). Scholars who focus on inclusion (Lähdesmäki et al., 2016) and exclusion (Antonsich, 2010) point to the discomfort, estrangement, and panic of not belonging (May, 2011). Such discussions of ‘non-belonging’ (Lähdesmäki et al., 2016) are highly applicable to the regional and remote, low-SES, mature-aged students that we explore in this article, yet ignore the ways (and places) that such students *do* belong. We posit that too much of the higher education literature to date asks how academics and higher education professionals might encourage students to adapt to and feel a sense of belonging on urban campuses, and that it does so at the expense of harnessing existing connections within local communities.

## Research design and methods

The Tasmanian findings reported in this article were one component of a larger research project, which investigated access to higher education for mature-age students in low SES, regional and remote communities in three Australian states (Relf et al., 2018). It followed a mixed-methods design (Creswell, 2014), with quantitative and qualitative approaches. The project received Social Sciences Human Research Ethics approval, and data were collected from cross-sectional surveys and semi-structured interviews.

This article focuses specifically on interview data collected from low SES, regional and remote communities in Tasmania. We analyse the data from the perspective of what works or is needed (or does not work) for people living, working, and studying in low SES, regional and remote Tasmanian communities, rather than focusing on what needs to change to fit the norms and expectations of ‘belonging’ in/to city campuses. These considerations are informed by Roberts’ (2014) ‘rural standpoint’ epistemology and our understanding of distance as something that should not only be discussed in relation to urban centres.

Interviews were conducted with community members/prospective mature-aged students; current mature-aged university students; and local stakeholders who worked or volunteered in roles related to education. The student interviewees were recruited through online surveys distributed to students currently or formerly enrolled in UTas’s University Preparation Program who met the inclusion criteria of being mature-aged and were living in a low SES and regional or remote area; survey respondents could indicate their interest in being interviewed upon completion of the survey. Community members/prospective students were recruited through local community events (where attendees were asked to complete a hardcopy survey and invited to express interest in being interviewed). The community events attended by members of the research team were in low SES communities; these communities were also in outer regional areas, which were surrounded by other outer regional or remote areas. Members of the research team attended four day-long or evening events in different parts of Tasmania. The stakeholder interviewees were recruited through their local workplaces and professional links to UTas.

The article’s first author worked in UTas’s University Preparation Program for a number of years; in addition, one of the members of the research team had extensive connections with local stakeholders from prior outreach and equity programs conducted at the university. A mix of approaches – face-to-face ‘in community’; hardcopy and online surveys; and telephone interviews – were undertaken for recruitment and data collection. This combination helped mitigate the challenges of ‘distance’, time, and financial costs when undertaking such research.

The findings reported include the qualitative analysis of 19 telephone interviews from the aforementioned participant groups. In discussing our findings, we focus largely on the accounts of the nine stakeholder interviewees, with supporting data provided by community members and students. The stakeholder participants worked or volunteered in local public libraries (Libraries Tasmania, n.d.), neighbourhood houses (Neighbourhood Houses Tasmania, n.d.),^6^ and trade trading centres^7^ (Tasmanian Government Department of Education, n.d.). Their roles and levels varied, and included centre managers, literacy coordinators, and staff in customer service roles, as well as volunteers. These stakeholders had particular insights into the services and facilities that locals could access while studying (or considering studying) at universities, the challenges students faced, knowledge of financial and resource constraints in higher education and their sectors, and often also had experiences of studying remotely online. Sampling was based on convenience, but sought interviewees of different genders, locations, and roles (see Table 1).

**Table 1:** Interviewee characteristics

Pseudonym	ASGS Remoteness Area*	Socio-economic status (SES) (SEIFA IEO*)	Role
Nick	RA2	low	Community member
Joel	RA3	low	Community member
Rebecca	RA3	low	Community member
Jade	RA2	low	Community member
Hayley	RA3	medium surrounded by low	Community member
Dorothy	RA2	low	Community member
Beattie	RA3	low	Community member
Judy	RA3	low	Stakeholder
Michelle	RA3	low	Stakeholder
Stuart	RA3	low	Stakeholder
Wendy	RA3	low	Stakeholder
Neil	RA3	low	Stakeholder
Liza	RA3	low	Stakeholder
Nadia	RA3	low	Stakeholder
Joanna	RA3	low	Stakeholder
Peggy	RA3	low	Stakeholder
Darren	RA2	low	Student
Robert	RA2	low	Student
Monica	RA2	low	Student

* Refer to 'Defining the terms' and footnote 1 for explanations of ASGS Remoteness categories and SEIFA IEO

The interviews were recorded and transcribed; the transcripts were analysed thematically (Braun & Clarke, 2022). Whilst NVivo software was used as a tool to assist with coding the data, the process of analysis was iterative and reflexive. It was also subjective, with the two authors making meaning of the data from their multiple perspectives as researchers, teachers, and equity practitioners in higher education. The two authors were located on opposite sides of the country, one familiar with the local context and the other not; we found our ‘emic’ and ‘etic’ perspectives useful in both the analysis and writing process as they encouraged further questioning, reflection, and clarification. Pseudonyms were assigned to interviewees to preserve their anonymity.

## Findings

Across the low SES, regional and remote Tasmanian communities in this study, mature-aged people encountered multiple challenges in embarking upon and participating in university studies, many of which related to their experiences of (not) belonging and distance. Practical impediments included minimal time, transport difficulties, and poor internet access. Some obstacles were based on universities’ practices, such as difficulties dealing with the mode of course delivery and a perceived lack of flexibility from teaching staff (lecturers/tutors). Physical distance to a campus was both a practical and financial obstacle, and impacted people’s senses of ‘not belonging’ in those spaces. Interviewees also proposed potential strategies, which included physical study spaces in their local areas, support from local people, and face-to-face study groups, which took advantage of their senses of local belonging.

### Experiences online and on campus: skills, time, course design, and funding

For students who cannot attend campus due to work, family, travel, or financial reasons, studying online is often presented as a realistic alternative by universities. However, interviewees highlighted challenges with studying online from their ‘distant’ local communities (see also Crawford & McKenzie 2011).^8^ One of the stakeholder interviewees, Michelle, who managed vocational education and training programs in a low SES, outer regional community, pointed out that not all mature-aged students had the computer skills required for online learning, and that ‘just throw[ing] it all online’ was not a solution ‘for the regional areas’. Monica, a student who had undertaken her degree largely online, reflected:

I wouldn’t study online [again]. I think online study takes too much time. Perhaps for younger people, but I can’t see any sense in studying if you don’t understand… I don’t know what it’s like now, but I think perhaps there is more help now, and I think they’re trying to do more, but once again they can only do so much… I don’t really agree with online learning because I think quite a few students see that [as] the easy way out, and it’s not.

A stakeholder interviewee, Peggy, who ran literacy programs for adults in a low SES, outer regional community, made a similar point about the assumption that placing courses online provides access to all: ‘initially when online learning came on everyone went, “that’s so great, everyone has access to everything”, but by golly it’s tough going’. From her own attempts at online study, she appreciated ‘the resilience’, ‘time management’, and ‘range of skills’ needed to do well. Peggy also elaborated on the isolation of studying online, and the persistence required by students. Online study could exacerbate distance between regional and remote students and their metropolitan-based peers, and fostered experiences of exclusion, non-belonging, and ‘distance from’ major campuses.

Although many students studied solely online, others needed to travel to urban campuses for their degrees, which involved travelling anywhere from 45 minutes to four-to-five hours one-way. Michelle also shared her experiences of having attempted university:

I actually started university a couple of times… and as a mature student, so I was actually working full time so it was just never really something that I could actually fit in. So I’d start something, probably do a couple of units, and then that would be the end of that.

With full-time work, it was unrealistic for Michelle to travel for her studies. She also reflected on positive university experiences, which included courses (units or modules) that catered for ‘time-poor’ students, such as a learning experience where ‘it was all around weekend workshop-based work, and online work, you know, assignments, projects-based work that we could all manage, in amongst our busy family and work lives’. In this example, the Course/Unit Coordinator had designed the content and assessments to align with the needs of students living far from the ‘main’ campus, as well as those engaged in full-time work. Michelle praised the thoughtfulness of the Coordinator responsible for this ‘flexible’ delivery, noting that it was an exception and that some teaching staff ‘are just plain inflexible’. Throughout her degree, she encountered difficulties with distance and her related inability to attend classes, as she was expected to travel to Hobart, a five-hour one-way drive. As a result, she did not complete the course.

Overall, stakeholders and students complained that course design tended to be inflexible for students learning ‘at a distance’, presuming that students were available on weekdays and able to travel to campuses. This perceived inflexibility was not necessarily due to academics’ lack of awareness; rather, structural and financial factors impacted their abilities to take a more regional/remote-centred approach. One factor informing ‘inflexible’ course delivery is the casualisation of the university workforce. Increasingly, university teaching staff are employed on short-term, precarious contracts, and are underpaid for this work (McKenzie, 2017, 2021) making it difficult to offer more expensive extra or repeat classes on weekends, or to provide off campus student support. As a result, students either experienced a sense of isolation and non-belonging through their participation in online courses, or faced exclusion and limitations in being unable to access face-to-face classes, a less likely experience for their peers who lived closer to campuses.

Time-limited grants and funding occasionally enabled initiatives that took a rural standpoint, but they lacked sustainability. Michelle noted the resultant instability of support programs in her low SES, outer regional area:

It seems to be there’s a bucket of money identified, so we’ll go hell for leather to form these connections, and then it just wanes then until the next bucket of money comes along. And I suppose that is the challenge… the ongoing presence.

She noted previous university widening participation and equity initiatives that had worked well in her community, but then vanished due to short-term funding or staff moving on to other positions, making it difficult to offer long-term support to students studying in regional and remote areas. Meanwhile, more long-term funding was directed to cheaper campus-centred initiatives, reinforcing the sense of distance, exclusion, and non-belonging experienced by regional and remote students.

### Distance to campus: financial, cultural, and geographical barriers

Students faced a number of other challenges related to travel and distance, with finance being a key barrier. This aligns with other research on equity-group and mature-aged cohorts (Devlin & McKay, 2017; Tones et al., 2009). Stakeholders and students noted the financial burden of travelling to campuses due to the cost of fuel and public transport. Transportation services were infrequent and not realistic for students who needed to take children to school before attending classes. Nadia, a client service officer in a small local library, noted the barrier of distance, pointing out that the often-proposed solution of carpooling for the two-hour drive to the closest campus was unrealistic for people in her community, who needed to coordinate different university schedules, alongside part-time jobs and family.

Similarly, when asked what the main obstacles were to studying at university, local community member Nick reflected on his own past decision to study at a teachers’ college after completing high school, rather than attending a (more distant) prestigious university he had been accepted to without financial support. He added that today, people in his local area faced similar problems:

Probably the idea of going away and leaving the community… living somewhere else [is the main problem]. They don’t know how they’re going to survive… How are we going to live and what sort of jobs are we going to do and all that sort of thing?

Other place-based barriers included feelings of discomfort and not fitting in at university campuses. Stuart, who managed a local library, described the strong sense of community in his rural area, noting that visiting spaces outside this locale could be confronting:

I think for a lot of people there is a strong sense of local community, and I think some of it is that people feel very comfortable within their own sphere, within their own sort of small community, but they feel uncomfortable going out of that… it [the university campus] is only half an hour, 45 minutes up the road. For some people that’s almost like another world.

Whilst the drive to the city campus might not be considered long in terms of time or kilometres travelled, particularly by Australian standards, it was nevertheless seen as considerable by some community members. Places were considered distant in terms of people’s behaviour, knowledge, expectations, and values. Stuart reflected on local community members whose families had lived in the small surrounding towns and rural areas for several generations. They found visits or moves to larger towns to be ‘outside their comfort zone’. This discomfort was about physical distance and ‘cultural’ difference, and was evident in people’s feelings of disconnection and ‘non-belonging’ on urban university campuses, in contrast to how they experienced their home communities.

Stuart mentioned a physical landmark between his rural community and the urban campus, remarking that ‘the big saddle’ is ‘almost a geographical barrier’. He added: ‘It’s a big deal almost for some people to cross over the saddle because you’re leaving… your home community and you’re going into Hobart’. This geographical landmark appeared to signal a boundary between ‘belonging’ and ‘not belonging’. ‘Distance’ was not only a matter of time travelled, but was also judged in terms of the terrain and conditions of that journey. Tasmania’s unpredictable and often severe weather conditions meant driving-related challenges, such as black ice, as noted by Stuart. This added to the difficulties of travelling to campuses, which not only felt ‘like another world’ – one where regional and remote students did not feel ‘at ease’ with themselves or their surroundings (May, 2011) – but could be challenging to reach. This highlighted potential students’ perceptions of place-based non-belonging on university campuses.

Feelings of disconnection, non-belonging, and distance to urban centres also impacted whether students even contemplated further studies. Judy, who managed a neighbourhood house, pointed out that people in her outer regional area (and neighbouring remote areas) did not see themselves in the university’s advertising:

[T]hese brochures that come out, about the higher education training… They don’t reflect our community… And I looked at the front brochure… all very nicely dressed, and I can tell you straight away, that my clients would look at that and that is not… that does not resonate with them because it’s not… that’s sort of like American TV to them. So straight away that’s a barrier that I see to engaging.

For local people to consider university studies, Judy asserted that they need to see that people ‘like them’ attended university; they also need to have social contact with and receive academic advice, support, and mentorship from people ‘like them’, such as those living nearby with whom they felt there was less ‘distance’.

Feelings of ‘not belonging’ were also connected to potential students’ limited knowledge about the possibility of higher education. Students appeared unaware of the local support already available, with Monica responding, when she was asked if she had access to any local support:

Well, the libraries, I think they have – or there is TAFE of course – but at the library, they have computer training and I think… but I really don’t know… I found that the only place I would do this sort of study is at a university.

Several of the stakeholder interviewees noted a lack of awareness of study opportunities and local study support in their communities. Peggy’s frustration was evident throughout her interview: ‘I would guarantee that, you know, we’re doing our best to promote what we have got, that most people wouldn’t have any idea, wouldn’t even think that they could go to the library to get help for their university studies’. Stakeholders like Peggy felt that community members were not taking advantage of local initiatives. However, as Judy highlighted, such initiatives were not necessarily well publicised, nor promoted effectively to potential mature-aged students in these communities. Overall, there were numerous barriers for students relating to ‘distance’ that intersected with students’ sense that they did not belong at university, including the cost or difficulties of travel and a sense of being different and distant to other university students. Meanwhile, they had little knowledge of the local support that might foster their sense of ease and belonging among other regional and remote, mature-aged, low SES students.

### Local support: physical spaces and local engagement

The findings above suggest that local spaces and connections have a significant role to play in engaging and fostering belonging among regional and remote students. Several of the communities explored here had been providing study facilities, as well as academic and social/emotional support from staff and volunteers, for a number of years. Local libraries in low SES, outer regional areas in Tasmania provided computer and internet access, as well as free printing facilities and quiet rooms for study (as described by all of the library stakeholder interviewees). A local trade training centre, situated alongside a secondary school, provided after-hours access to computers and internet in a computer lab (as noted by Michelle). Such physical spaces were invaluable for students who required reliable internet and access to free printing, and aimed to combat experiences of exclusion, distance, and missed social and educational opportunities (Gore et al., 2019; Webb et al., 2015), as well as providing academic support.

These facilities were especially valuable in light of the poor internet quality across much of regional and remote Australia. Several interviewees noted universities’ assumptions that students had access to the internet and computers, and digital literacy, a finding that accords with previous studies (Crawford, 2021; Crawford & McKenzie, 2011; Devlin & McKay, 2017; Nelson et al., 2017; Pollard, 2018; Thomas et al., 2020). Michelle mentioned making a computer lab in their vocational educational facility available to university students:

Then people could come in and actually do stuff, print stuff out if they need to, and those sorts of things in an environment where their computer’s not going to die… or you know, the line’s not going to drop out halfway through uploading their assignment.

Michelle spoke about a university student who had completed her Education degree online, regularly using the local facility during her studies. This seemingly ‘small’ service had made an important practical contribution, working to replace some of the services that were more easily accessible to students in more populated areas or who lived close to their campuses.

In addition to the practical support provided by local libraries, trade training centres, and neighbourhood houses, their staff and volunteers provided support with using technology, finding information, referencing, and advice on academic literacies and study skills. Neil, a volunteer facilitator of a weekly study group, described how students could drop in, work on their assignments, and debrief with each other about their experiences. A retired academic, self-described life-long learner, and current student, Neil would model ‘being a student’ during these study sessions. Mentors and/or study group facilitators like Neil encouraged a sense of community among students, as well as working to minimise some of the ways students could feel ‘culturally distant’ from their universities and peers, particularly given the types of learning they had been able to access in the past. A similar initiative was also mentioned by Michelle, in relation to her experience of a study group in a different community, which also had a local mentor who understood the academic culture and expectations of universities. As Michelle, reflected:

We actually had a study group, and, again, the university had a mentor here, who actually worked with a study group, so we sort of… I think they sort of pulled together students studying in a variety, range of areas, and we made our lab, our computer lab, available to that group after hours and, again, that was another sort of initiative, but they [these sorts of schemes] sort of come and go.

Students received guidance and support in study groups (referred to as ‘the study circle’ in one community) at their local libraries. Peggy explained that study groups had been a semi-regular fixture for many years, depending on interest:

We just had a regular time basically, and we would address people’s questions and just help them as needed. We weren’t there to teach as such, but it really was a support role, so supporting them with the [Learning Management System], supporting them with the process. If they’re thinking, “Oh no, my assignment is going to be late, what do I do?” so helping them with who they need to call for that.

Peggy noted the benefits for online students who, faced with feeling isolated and distant from their campuses, ‘almost without fail they said without this study circle we would not have done it’.

Membership of study groups often consisted of students in UTas’s University Preparation Program, due to a collaboration initiated by the UPP Manager in 2013 with Libraries Tasmania (Jarvis et al., 2013). The study groups were facilitated by local community volunteers who received training, resources, and support from the UPP Manager. Interviewees highlighted the number of willing local volunteers – typically retirees who had relocated to the communities – who possessed a thorough understanding of university ‘culture’ and expectations from their prior experience as academics and/or lifelong learners (such as Neil, a volunteer in his local library, mentioned previously). In one small low SES, outer regional community, Judy noted that the neighbourhood house she managed had 140 volunteers, emphasising how driven the community was. However, the reliance on one paid staff member – the UPP Manager – for oversight, coordination, and support across the state made it difficult to sustain this initiative. When this staff member moved, the initiative lost momentum, thus showing the ripple effect of the insecurities of university employment. Initiatives with the potential to harness and build a sense of belonging among regional and remote students were regularly stymied in this way.

## Discussion

A particular challenge of regional and remote students is their feelings of distance, discomfort, and ‘not-belonging’ in universities. Yet focusing on the ways that these students are excluded, isolated, and ‘distant from’ more populated areas also serves to problematise these students. By applying the central concept of belonging while adopting a rural standpoint, clear opportunities emerge for establishing or expanding coordinated networks of regional and remote study spaces, peopled by locals and centred on making students feel they belong ‘at’ university (wherever that might be). We elaborate on the ‘untapped’ potential of local people and spaces in regional and remote higher education, while considering the sustainability of community collaborations and funding arrangements.

### Belonging in place: utilising local spaces and people

Our findings highlight practical, geographical, cultural, and financial challenges of university study faced by current and prospective mature-aged university students in low SES, regional and remote areas in Tasmania. Studying online is often presented as a viable alternative for people who juggle work and families, and do not have the time or finances for frequent travel to attend classes. However, online study also has its challenges, such as unreliable internet; unpreparedness for this mode of study; missed learning, career, and social opportunities; and isolation from peers and staff (Gore et al., 2019; Webb et al., 2015).

Our findings highlight the sense of isolation, distance, unease, and non-belonging experienced by regional and remote students in university spaces (Jury et al., 2017; May, 2011). Interviewees argued that students felt they did not belong on campus, and did not see themselves as being like other students. This was exacerbated by a sense of geographical separation. Even when distances were not large, local landmarks, unpredictable weather patterns, and expensive and time-consuming travel made them seem so (Currie et al., 2007; Kenyon, 2011). Here, Kenyon’s (2011) findings are of interest, as she draws links between transport difficulties and social exclusion in higher education. She sees universities as inaccessible spaces: geographically and in other ways (Gore et al., 2019; Webb et al., 2015).

Previous approaches to student ‘non-belonging’ have sought to better integrate students into urban campus spaces. These approaches tend to problematise students’ feelings of unease with and distance to/from campuses, as well as ignoring the possibilities of local belonging, some of which are already apparent. At present, if students have access to a regional (satellite) campus or a regional study hub (e.g., a Regional University Centre), they can complement their online studies with time spent at a physical space, benefiting from practical, academic, and emotional support from staff and peers (Crawford, 2021; Davis & Taylor, 2019; Rossi & Goglio, 2020). While such spaces increasingly provide support for local students in inner regional areas, they are less accessible for students in more sparsely populated outer regional, remote, or very remote areas. We have found that this gap can be filled by using existing local spaces, such as libraries and schools, some of which are already being used by university students, and where academic support is in some cases being provided by staff and volunteers.

In considering regional and remote students’ educational needs, we suggest a place-based model suited to the needs and availability of local communities: one that does not see students as straightforwardly ‘distant from’ the campus. We propose an expanded, coordinated, and funded network of local study places and local people, including libraries, schools, neighbourhood houses, and vocational and further education spaces, in addition to, and complementing, regional (satellite) campuses and Regional University Centres. In this network of physical spaces, online students can be supported, connected, and engaged in their studies whilst located in their regional and remote communities.

### ‘Untapped’ potential: local people and spaces

The value of local infrastructure has been acknowledged in relation to Australian regional and remote education. In a review of Australian higher education more than a decade ago, Bradley et al. (2008, p. 111) proposed partnerships between higher education and communities, local schools, businesses, industry, and other sectors. Using schools as ‘community hubs’ has been suggested by Gore et al. (2019, p. 66), as it allows ‘community members to utilise internet access or videoconferencing facilities to complete courses via online mode with the support of peers’. Others have explored collaborations between universities and public libraries to support university students studying online in low SES, regional and remote areas (Power et al., 2019).

Our findings highlight the need and potential of existing local infrastructure. Through looking beyond the metropolitan norm and adopting a ‘rural standpoint’ (Roberts, 2014), it becomes clear that feelings of regional and remote belonging can and should be harnessed. This lies in contrast to the ways in which low SES, mature-aged, regional and remote students are often treated, as lacking or problematic (Jury et al., 2017; Regional Education Expert Advisory Group, 2019). We thus propose a strong emphasis on initiatives that build on local regional and remote resources and facilities, as well as local people’s skills, knowledges, and experiences of university education (including past experiences of non-belonging, isolation, distance, and exclusion). For instance, local libraries and schools can be utilised as study hubs for mature-aged students in regional and remote places, offering ‘drop-in’ opportunities and other useful supports to mitigate problems that students might face. Our localised approach extends previous studies, which document the barriers of geographical distance, expensive or limited transportation, and little access to face-to-face social and career opportunities (Currie et al., 2007; Kenyon, 2011).

Many regional and remote communities already tap into the knowledges and educational experiences of retirees and people relocating to their communities who, like Neil previously mentioned, are often eager to support education in their local area. The Tasmanian regional and remote student experience is regularly characterised by social isolation from peers and a sense of distance, a finding that mirrors previous studies (Currie et al., 2007; Kenyon, 2011). Our findings suggest that local facilities, resources, and people remain a largely untapped resource, which can help mitigate some of the perceived distance and isolation experienced by students. Such initiatives, which involve staff, volunteers, and local spaces, require a coordinated approach with training, support, and resources. While we are not proposing that library staff and volunteers be expected to take on the core business of academic staff and universities, volunteers in many of the programs discussed above were trained in how to facilitate study groups, provided with resources, and had a contact person with whom to troubleshoot. At a minimum, sustained funding is required for the overarching coordination of any such initiative. This should not be treated as ‘additional’ funding, but (in accordance with taking a rural standpoint) as resourcing that is central to the functioning of universities, which are populated not only by urban students but by regional and remote students.

### Community collaborations: long-term funding for support and adult outreach

There is thus a need for long-term and place-based approaches to resourcing local study support in regional and remote areas. At present, the disappearance of previously successful programs – due to staff changes and short-term funding – appears to be commonplace. While funding is important for starting and maintaining initiatives, and paying staff, much of the necessary infrastructure and people are already in place. What is required is a long-term commitment – collaborations between universities, the vocational education and training sector, libraries, and communities – to coordinate, maintain, and sustain projects and networks. Certainty and continuity of funding for regional initiatives that centre local belonging and solutions, and moving beyond an approach that problematises their ‘distance from’ urban centres, is crucial. One of the key issues identified by interviewees was that local initiatives are intermittent, emerging and disappearing with changes in funding and student numbers. Resources become scarce and organisers move on. It is important to maintain the momentum in these initiatives, as re-starting previous projects can be costly and lead to lost knowledge and expertise. Funding for local study support – such as for coordinating a network of volunteers in local places – would not only benefit individual students and their communities, but also universities in terms of improved retention and completion rates and the resultant funding received.

Moreover, initiatives need to be suited to and draw on existing social connections within their local contexts (Lähdesmäki et al., 2016). For instance, communities with larger numbers of students may benefit from opportunities to study and socialise with other students in their communities, fostering a sense of belonging *off* campus with regional and remote peers. Others may benefit from intensive ‘adult outreach’ (see also Bradley et al., 2008). Universities currently undertake outreach with regional and remote schools, targeting school leavers. However, ‘adult outreach’ creates an awareness of possibilities and encourages mature-aged people to consider university studies for themselves and others. Having a local, physical presence – with permanent banners and brochures as well as temporary public ‘campuses’ where students can connect and ask questions – may combat community members’ lack of awareness of their study options. Such outreach could be undertaken at regional and remote libraries, community centres, or public events.

## Conclusions

For mature-aged students who live, work, and study in regional or remote areas, relocating to study on campus is not, in most cases, a realistic option. Online study is one alternative, but it has well-documented challenges, such as isolation, exclusion, and non-belonging. These tend to be the focus of much research, yet emphasising the distance between regional and remote students and their on-campus counterparts often serves to hold up the latter’s experiences as something to be emulated (Jury et al., 2017; Regional Education Expert Advisory Group, 2019). However, interviewees in this Tasmanian study described existing physical places, such as local public libraries, as providing spaces and support for students within their regional or remote communities, where they could study, and use computers and the internet. Local people – library staff, volunteers, and students’ peers – provided social connections and academic support via joint initiatives between, for example, universities and libraries. Yet such schemes, which have the potential to foster a sense of belonging ‘at’ university from within a student’s local area, were intermittent and not always well funded or staffed. A long-term funded, localised approach to learning support – one that takes a rural standpoint and centres regional and remote (local belonging) rather than urban belonging – is crucial in ensuring that students are able to engage and succeed in their studies, regardless of their geographical location, SES, or age. University policies and funding models need to move beyond their current urban standpoints, and foster access, participation, and belonging by taking more localised approaches.

## Data Availability

Data was collected under the condition of anonymity and confidentiality, and is therefore not available to be accessed.

## Footnotes

1 As described by the Australian Bureau of Statistics (2018b, p. 7), scores incorporate ‘educational and occupational aspects of relative socio-economic advantage and disadvantage’. This includes formal qualifications and skills that are required to perform different occupations. Low scores indicate high proportions of people without qualifications, jobs, or in low skilled employment.

2 While we mostly use the term ‘regional and remote’ in this article, more specific categories such as ‘outer regional’ or ‘remote’ will be used in the findings when reporting on participants. The term ‘rural’ is used when it is referenced by a participant or in the literature and more accurately evokes a participant’s experience.

3 The six equity groups include students that ‘are from non-English speaking backgrounds (NESB); have a disability; are women in non-traditional areas; identify as [I]ndigenous; are from low SES (socioeconomic status) locations based on postcode of permanent home address; and are from regional and remote locations based on postcode of permanent home residence’ (Department of Education, Skills and Employment, 2020a).

4 In Australia, the proportion of ‘mature-aged students’ ranges from approximately 30 per cent (for students commencing aged 26 or older) to 60 per cent (commencing aged 21 or older) of the regional and remote cohort (Crawford, 2021, p. 6).

5 The University Preparation Program is UTas’s ‘enabling education’ course; such courses are also referred to as bridging courses, transition, or access programs.

6 Neighbourhood houses are community-based organisations that respond to the needs of their community; they are also known as community centres/houses or learning centres.

7 Trade training centres are specialised facilities established in regional locations that enable school students and adult community members to undertake accredited training in the trades.

8 Although the data were collected well before the COVID-19 pandemic, the findings have implications for the emergency shift to online learning, as well as for online delivery and learning in post-COVID-19 times.
